# Investigations on Inhibitors of Hedgehog Signal Pathway: A Quantitative Structure-Activity Relationship Study

**DOI:** 10.3390/ijms12053018

**Published:** 2011-05-11

**Authors:** Ruixin Zhu, Qi Liu, Jian Tang, Huiliang Li, Zhiwei Cao

**Affiliations:** 1 Department of Bioinformatics, School of Life Sciences and Technology, Tongji University, 1239 Siping Road, Shanghai 200092, China; E-Mails: rxzhu@tongji.edu.cn (R.Z.); qiliu@tongji.edu.cn (Q.L.); 2 Department of Natural Medicinal Chemistry, School of Pharmacy, Second Military Medical University, Shanghai 200433, China; E-Mail: tangjian-sh@sohu.com

**Keywords:** QSAR, Hedgehog signal pathway, inhibitor, cyclopamine

## Abstract

The hedgehog signal pathway is an essential agent in developmental patterning, wherein the local concentration of the Hedgehog morphogens directs cellular differentiation and expansion. Furthermore, the Hedgehog pathway has been implicated in tumor/stromal interaction and cancer stem cell. Nowadays searching novel inhibitors for Hedgehog Signal Pathway is drawing much more attention by biological, chemical and pharmological scientists. In our study, a solid computational model is proposed which incorporates various statistical analysis methods to perform a Quantitative Structure-Activity Relationship (QSAR) study on the inhibitors of Hedgehog signaling. The whole QSAR data contain 93 cyclopamine derivatives as well as their activities against four different cell lines (NCI-H446, BxPC-3, SW1990 and NCI-H157). Our extensive testing indicated that the binary classification model is a better choice for building the QSAR model of inhibitors of Hedgehog signaling compared with other statistical methods and the corresponding *in silico* analysis provides three possible ways to improve the activity of inhibitors by demethylation, methylation and hydroxylation at specific positions of the compound scaffold respectively. From these, demethylation is the best choice for inhibitor structure modifications. Our investigation also revealed that NCI-H466 served as the best cell line for testing the activities of inhibitors of Hedgehog signal pathway among others.

## 1. Introduction

The hedgehog signaling pathway plays a key role in the control of cell differentiation, growth, and proliferation [1]. Briefly, hedgehog signal pathway is composed of four important components including Sonic Hedgehog, Patched, Smoothened and Gli transcription factors. Sonic Hedgehog is a secreted protein that can transduce signals between cells. Patched acts as a receptor protein to be binded by Sonic Hedgehog. When Sonic Hedgehog is absent, Patched can block the function of Smoothened. In addition, Smoothened would be activated and initiate a signaling cascade that results in the activation of Gli transcription factors when Sonic Hedgehog binds with Patched. These Gli transcription factors will translocate into the nucleus where the transcription of target genes is controlled. Recent studies have found that constitutively activating the pathway can trigger cancer in adult humans, leading to basal cell carcinoma, medulloblastoma, rhabdomyosarcoma, prostate, pancreatic and breast cancers [2–5].

Due to the direct relationship between the activation of hedgehog signaling pathway and oncogenesis, cancer researchers have been dedicated to find specific inhibitors of hedgehog signaling since it will provide efficient therapies for a wide range of malignancies [6–8]. Until now, only specific Smoothened inhibitors have been identified. Cyclopamine, a steroid alkaloid isolated from the corn lily (Veratrum californicum), is one of the small chemical compounds that specifically inhibit Smoothened in the hedgehog signaling pathway [9]. However, there is still no efficient pathway to synthesis Cyclopamine because of its low solubility in aqueous or polar solvents and little effort has been devoted into the synthesis of cyclopamine derivatives [10–13]. In order to develop clinically effective drugs, modifications of parent lead compounds to generate derivatives to study the structure-activity relationship (SAR) become necessary [13]. Janardanannair *et al.* [9,14] have pioneered such investigations on the SAR of cyclopamine derivatives. Their results quantitatively indicated that modification on secondary amine and oxidation to ketone from 3-Hydroxy could help to influence the activities of cyclopamine derivatives. However, both studies had less than 30 samples, which is far from satisfactory for a sound QSAR study.

In order to better understand Hedgehog signal pathway as well as design efficient inhibitors for this pathway, 93 cyclopamine derivatives were synthesized and their activities were tested against four different cell lines (BxPC-3, NCI-H446, SW1990 and NCI-H157) respectively [15,16]. Based on these experimental data, a systematical investigation was carried out on SAR of inhibitors of Hedgehog signal pathway by incorporation of various statistic modeling approaches and comparison of different descriptors and statistical division approaches of these data.

## 2. Results and Discussion

Based on the computational framework outlined in Material and Methods, the following results or clues were obtained for the QSAR modeling of inhibitors of Hedgehog signal pathway.

### 2.1. The Influence of Descriptors on the QSAR Modeling of Inhibitors of Hedgehog Signal Pathway

As mentioned above, two distinct sets of descriptors were tested to describe the 93 chemical compounds respectively (Table 1 and Table 2). For the self-fitting of training data (highlighted in red), we found that the models derived from physical properties are more efficient than those derived from topological indices for QSAR modeling. It can be seen that almost all the values of σ in this case are negative. However, with regard to independent testing (highlighted in royal blue), it seems that QSAR models derived from the DLI descriptors [17] are much more robust than those derived from general descriptors [18], and in this case almost all the values σ are positive. As an intermediate state, the values of σ derived from cross validation (highlighted in yellow-green) contain several negative and positive ones respectively. In total, the above mentioned result indicated that when projecting the connection table information into physical properties, the general descriptors will lose some structural information of a compound. Such loss of information is different for training and testing datasets since this information is highly dependent on the conformation and structural essence of a molecule.

In conclusion, models derived from DLI are much more stable for both training data and testing data, while general descriptors cannot guarantee such stability and scale in independent data.

### 2.2. The Influence of Data Division on the QSAR Modeling of Inhibitors of Hedgehog Signal Pathway

It is normally known that QSAR predictions are only reliable within or near the property space used to train the model. Preparing a robust, unbiased and sufficiently large training set is critically important for the building of a proper statistical model. As mentioned above, two data division methods, *i.e.*, Diverse Subset and Cluster plus Diverse Set were applied to divide our dataset into training set and testing set.

In order to statistically reveal the difference between the results influenced by two such kinds of data divisions, pair t-test was performed and the p-value derived from the above two tables (Table 1 and Table 2) was 0.88 (>0.05), which indicates that there is no significant statistical difference for these two data divisions for QSAR analysis. Our result has shown that clustering data before calculating the diverse set does not produce a significant influence on the QSAR models. This result was explained by analysis of the detailed algorithm in calculating the diverse set as follows: The Diverse Subset method used in MOE [19] ranks entries based on the whole dataset diversity, that is, the calculation of Diverse Subset itself is a global diversity comparison procedure. For the Cluster plus Diverse Set method, although an extra preprocess of clustering data exists, Diverse Subset still happens within every sub-cluster and the main difference, compared with the former, is that calculating diverse subset becomes a local procedure based on each clustering. It can be seen that essentially the two division methods have little influence on the final distribution of training data and testing data. Thus, as expected in our results, no significant differences for the results of these two division methods exist.

### 2.3. Comparison of PLS and SVR for QSAR Data Regression

When building a QSAR model, linear regression methods are normally preferred to the advanced non-linear methods, since the linear models are easier to use for a physical explanation of the prediction results. The most classical liner model in QSAR is PLS, which have been widely used in popular computer-aided drug design software [19–21]. In our study, PLS (MOE-PLS) was first chosen to derive our QSAR models. However, as indicated in Tables 1 and 2, this linear model failed to achieve satisfactory results in QSAR study. The correlation coefficients from self-fitting testing and cross validation testing are all less than 0.65.

**Figure 1.**
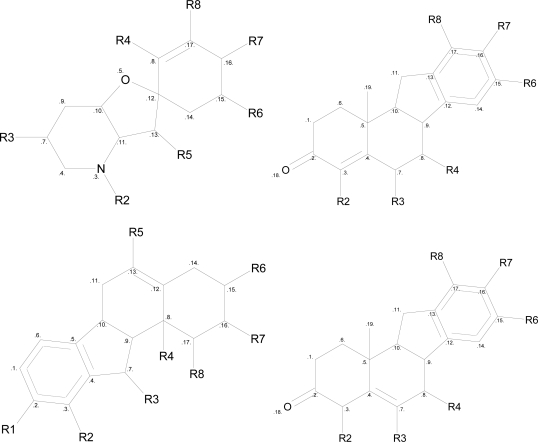
Four scaffolds found in our experimental data.

**Figure 2.**
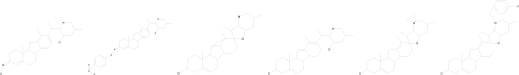
Six molecules that did not match any of the scaffolds, as mentioned above.

Since advanced machine learning methods such as ANN [22], Bayesian inference [23], Random Forest [24] and SVM [25] have been successfully applied in QSAR study [26–36], our QSAR models were rebuilt using the SVR method, which is a derived regression model with powerful fitting ability as well as excellent prediction accuracy [36–39]. In anticipating results, this method behaved well in the self-fitting testing of our training data (R2 is nearly 0.9) as well as in the cross-validation testing. Nevertheless, this method still performed badly in the independent test data, which indicates that such machine learning methods may not be generalized enough in the cyclopamine data. This is probably due to the fact that a substantial diversity exists in our dataset. Among the 93 data, four different scaffolds were found (Figure 1). In addition, there were still six molecules that did not match any of the scaffolds (Figure 2).

### 2.4. Comparison of Binary Bayesian Inference and SVM for QSAR Data Classification

When the qualities of the data or the underlying mechanism are not suitable for regression modeling, the binary classification was applied on the data to uncover their probabilities to be active or inactive. MOE has offered a binary filter to filtering the numerical data. Any properties which can be represented in a binary (yes/no) way (like active/inactive, toxic/non-toxic, drug-like/non-drug-like, permeable/non-permeable, *etc.*) could be mapped onto such a filter. Thus, the binary classification model was used to rebuild the QSAR models to further reveal their intrinsic characteristics. MOE’s binary filters (yes/no) are based on the Bayesian inference technique as mentioned in *Material and Methods*. Continuous activity data (non-binary) can be transferred to binary values with a specific threshold criterion. In our study the IC50 of the drug compound is used as a cut-off.

As shown in Table 1 and Table 2, the binary model behaved well on both training data and testing data sets. The overall prediction accuracy is improved to nearly 0.8 against NCI-H446 cell line. (Some were up to 0.906). This result has indicated that the binary QSAR classification model is more suitable to guide the direction of designing novel inhibitors of Hedgehog signal pathway.

The SVM classification was also applied to further validate the efficiency of binary classification models compared with regression models. The results shown in Table 1 and Table 2 reconfirmed that for our data the binary classification model is probably more suitable for QSAR analysis.

### 2.5. Cell Line Analysis

Four different cell lines (NCI-H446, NCI-H157, SW1990 and BxPC-3) were used to test the cytotoxicity of the 93 compounds. However, only the data of NCI-H446 can produce a reasonable model by QSAR analysis; the prediction accuracy of the models against all the other cell lines is about 0.6.

Why do some specific cell lines not fit well to our QSAR analysis? We speculate that the most likely reason is the non-specific cytotoxicity effect of these compounds to the other three cell lines. For example, HCI-H157 and BxPC-3 do not express the Gli and Smoothened protein, respectively [40,41]. That means that the cytotoxicity effect of these compounds may not directly result from the inhibition of hedgehog signaling. In addition, although sustained hedgehog signaling activity can be detected in SW1990 cells [41], it is very likely that cell lines grown *in vitro* may lose their dependence on hedgehog signaling for survival [42]. For example, the IC50 of positive compound (cyclopamine) is 9.13 μg/mL for NCI-H446, 38.11 μg/mL for BxPC-3, 61.05 μg/mL for SW1990 and 58.33 μg/mL for NCI-H157. That is to say, firstly, HCI-H466 cells were most sensitive to the hedgehog signaling inhibitor. In addition, the SW1990 possibly mutated and lost the hedgehog signaling in our experiment. In summary, the non-specific effects may result in the variance of the data of the cytotoxicity and finally affect the QSAR analysis.

### 2.6. Structure Activity Report

In our study, *SAReport* was applied to present a direct instruction on how to modify the structure of a compound and make it a better inhibitor of hedgehog signal pathway. All the structure modifications are listed in the supplementary material. Here the top three structures were selected with their activity improvements according to different modification mechanisms.

**Figure 3.**
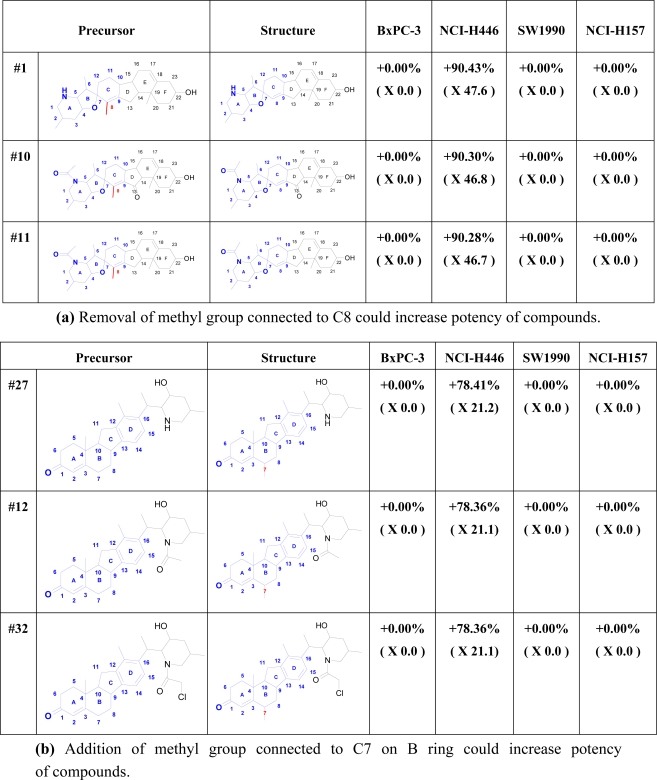

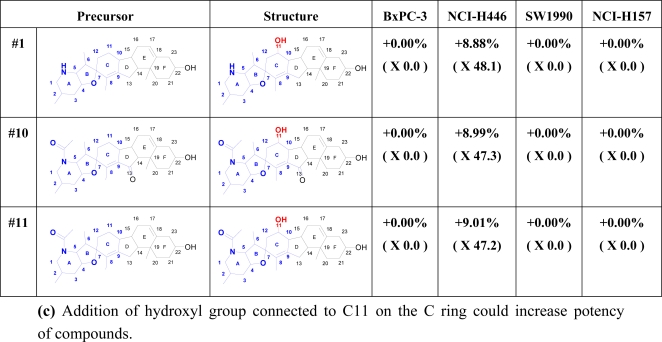
*SAReport* of Hedgehog inhibitors.

The first important finding is that through such *SAReport* we validated our former finding that only the data to cell line NCI-H446 can obtain a reasonable QSAR modeling result (indicated in Figure 3). Secondly, our *SAReport* has shown that demethylation, methylation and hydroxylation at a specific position of the inhibitor scaffold may highly improve their activity. As indicated in Figure 3, demethylation at position 8, methylation at position 7 and hydroxylation at position 11 provided three possible ways to improve the inhibitor’s activity. In addition, the *SAReport* shows that demethylation seems to be the most efficient approach to improve activity among others. This conclusion provides the first proven set of efficient inhibitor structure modification methods in order to improve their activities. All these results will definitely shed new light on the future work of inhibitor synthesis.

## 3. Material and Methods

**Figure 4.**
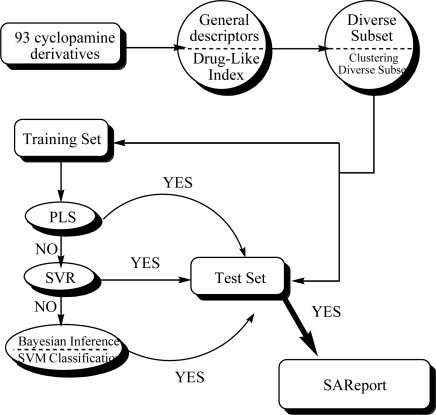
General computational workflow used in our study.

A comprehensive computational workflow was designed to perform QSAR analysis on the inhibitors of Hedgehog signaling. This workflow is outlined in Figure 4. Details are listed below.

Our analysis started by using two different descriptors, *i.e.*, general descriptors and drug-like index to describe the 93 cyclopamine derivates. In order to construct the training set and testing set for statistical modeling, two kinds of data division method were tried, *i.e.*, *Diverse Subset* and *Clustering Diverse Subset* for data generations. Then, based on the training data we obtained, different statistical modeling approaches including PLS, SVR, Naive Bayesian classification and SVM classification were applied to evaluate their abilities for QSAR modeling. It should be noted that the former two methods are used to perform regression on the QSAR data and the other two methods are focusing on data classification. These approaches were applied in the testing data for further validation and derive useful clues for the designing of efficient inhibitors of Hedgehog signal pathway. Finally a *SAReport* of QSAR modeling of such inhibitors was presented for the first time.

### 3.1. Dataset and Data Division Methods

93 cyclopamine derivatives together with their activities against four different cell lines (BxPC-3, NCI-H446, SW1990 and NCI-H157) were tested and are listed in the supplementary material.

Two different approaches were applied to divide these experimental data into training set and testing set for our following statistical modeling. Details followed.

#### 3.1.1. Diverse Subset

Briefly, the *Diverse Subset* method presented in MOE ranks compound entries based on diversity. In the procedure of data division, the first entry of the original dataset is taken as a reference and will always be viewed as part of a diverse subset. Then the most “distant” compound data is assigned #2, and then the most distant compound to these two is assigned #3 and so on until the required number of diverse compounds is identified or the whole dataset is ranked in diversity order. To determine which unranked entry is farthest from all already-ranked entries, the distance between each unranked entry and each ranked entry is calculated. For each unranked entry, the minimum of its distances to each ranked entry is found. The entry with the largest such “minimum distance” is deemed to be the farthest. Then such ranked dataset is divided into two parts as a training dataset (65% of the original set) and testing dataset (35% of the original set).

#### 3.1.2. Cluster plus Diverse Subset

Compared with the above method, a clustering process is used here before Diverse Subset. Then the Diverse Subset is performed on each cluster to rank them respectively. Finally the training dataset and testing dataset are generated by summarizing the sub-training dataset (65% of every sub-cluster dataset) and testing dataset (35% of the every sub-cluster dataset) from every sub-cluster, respectively. It should be noted that MOE can cluster the whole data based on the descriptors or fingerprints. For time purposes, the descriptor-based clustering in MOE was used in our study because it is a simple 3N algorithm whereas fingerprint-based clustering uses the N2 Jarvis-Patrick algorithm.

### 3.2. Structural Descriptors

There are lots of descriptors to describe a chemical compound, including constitutional descriptors, physiochemical property descriptors, electronic descriptors, topological indices, geometrical descriptors, and quantum chemistry descriptors, *etc.* However, no set of descriptors is capable of performing spectacularly better than the others. Thus, to build our QSAR model, the widely applicable set of descriptors, *i.e.*, the general descriptors was selected. Also, DLI descriptors was adopted for a complementary comparison.

General descriptors include atomic contributions to van der Waals surface area, log P (octanol/water), molar refractivity and partial charge. These descriptors are applied to the construction of QSAR models for boiling point, vapor pressure, free energy of salvation in water, solubility in water, thrombin/trypsin/factor Xa activity, blood-brain barrier permeability and compound classification. The wide applications of these descriptors have suggested their important usage in the QSAR modeling, combinatorial library design and molecular diversity work.

On the other hand, DLI descriptors acts as an approach to measure drug-like compounds, as first presented by Xu *et al.* Then it was used and modified as a set of descriptors by MOE. These descriptors characterized the hierarchy of drug structures in terms of rings, links, and molecular frameworks.

Although these two sets of descriptors are both computable from connection table information, they partly complement each other. Normally, general descriptors have a preference for physical prosperities of compounds, while DLI descriptors favor simple topological indices of compounds.

### 3.3. Statistic Modeling

In our computational framework, various statistical models were incorporated to evaluate their performance in QSAR analysis of inhibitors of Hedgehog signal pathway, and we wanted to find the most suitable statistical analysis method for the QSAR modeling of such data. Detailed descriptions of each statistical method are listed below.

#### 3.3.1. PLS Method

The PLS QSAR method [43,44] was widely employed in the study of QSAR modeling by the QuaSAR-Model module of MOE 2008. This is arguably the most traditional and least sophisticated QSAR approach among those explored in this study. It was explored here to test if it could build reliable models for underlying data sets using the simplest approach. In our study, we applied the PLS method presented in MOE and the number of components was set to no limit on the degree of the fit. The maximum condition number of the principal component transform of the correlation matrix *S*, the condition limit, was set at 1.0 × 10^6^ which is a very high setting. The leave-one-out cross validation (LOO-CV) scheme was used to validate the models and the correlation coefficient (Q2) and root-mean-square error (RMSE) were reported.

#### 3.3.2. SVR

SVR was used here to compare with PLS regression, which has proven to be a powerful regression technique in many applications. SVR is the regression version derived from SVM which was proposed in 1996 by Vladimir Vapnik *et al.* [45]. This regression method depends only on a subset of the training data and the cost function for building the model ignores any training data close to the model prediction (within a threshold *ɛ*). Intrinsically, SVR maintains all the main features that characterize the maximal margin algorithm and a non-linear function is learned by a linear learning machine in a kernel-induced feature space while the capacity of the system is controlled by a parameter that does not depend on the dimensionality of the space. In summary, the basic idea of SVR is to map the data into a high-dimensional feature space via nonlinear mapping, and perform linear regression in this space.

#### 3.3.3. Binary Bayesian Inference

The binary bayesian QSAR method was employed by using the QuaSAR-Model module of MOE 2008. In this modeling, the numerical values of inhibitor activity were transferred to binary classification labels, thus greatly reduced the noise of the data. That is, the binary model is used to predict a probability of a given compound to be either active or inactive rather than their numerical values. Since no quantitative estimation of the actual activity is derived, the compounds are referred to as “active” if its predicted probability of being active is more than 0.5.

In binary Bayesian inference for each compound, the following steps were applied to predict their probability of being active [46]:

- Estimates two distributions: one for the active compounds and one for the inactive ones in the training set. The separation of active and inactive sets is manually defined by a Binary Threshold.
- Counts the frequency of occurrence of a particular descriptor value in active and inactive cases.
- Accumulates a histogram of the observed sample values over the classes. The distribution is convoluted with a Gaussian (σ = 0.25, the smoothing width) to avoid sensitivity to bin boundaries.
- A histogram of property distributions is derived for each descriptor for “active ” and “inactive” (yes/no) sets. Those descriptors which differentiate the two sets will have a high impact in the model, those which do not, will drop out.

#### 3.3.4. SVM Classification

Compared with binary Bayesian classification, the SVM classification was also applied for our QSAR data. SVM works by mapping the training data into a feature space with the aid of a so-called kernel function and then separating the data using a large margin hyperplane. Intuitively, the kernel computes a similarity between two given examples. Most commonly used kernel functions are radial basis function kernels and was used in our experiments. SVM classifiers are generated by a two-step procedure: First, the sample data vectors are mapped (“projected”) to a very high-dimensional space. The dimension of this space is significantly larger than the dimension of the original data space. Then, the algorithm finds a hyperplane in this space with the largest margin separating classes of data. It was shown that classification accuracy usually depends only weakly on the specific projection, provided that the target space is sufficiently high dimensional. Sometimes it is not possible to find the separating hyperplane even in a very high-dimensional space. In this case a tradeoff is introduced between the size of the separating margin and penalties for every vector which is within the margin.

### 3.4. SAReport

SAReport [47] is an important tool for the visualization and analysis of project SAR data introduced by MOE recently. SAReport contains sophisticated analysis methods to help scientists identify important groups and make more effective choices for synthesis.

Briefly, the Suggestions table in *SAReport* consists of a list of hypothetical molecules, constructed from available pieces, which are predicted to have a high probability of activity. The pool of hypothetical molecules is prepared by enumerating all of the input molecules, and performing single-point mutations at each of the substitute positions, with each of the R-groups that have been observed in the equivalent position for some other molecule in the dataset. The unique list of chimerical molecules is then rated according to an estimate of probability, scaled and balanced to match the distribution of activities found in the input set. The scores are scaled in such a way that a value of 0 indicates that the hypothetical molecule is as likely to be active as an average molecule in the input set, while positive values are more likely. The chimerical molecules are ranked by their probability of activity, multiplied by a weighting factor, which is a measure of cumulative similarity to other molecules in the database. A higher weighting implies that a larger statistical base is available to make the prediction. The most promising candidates are listed first. The molecule from which the candidate was mutated is shown, along with its property information. The new structure is shown to the right, along with the prediction. The percentage value is the increased probability of activity, and the number in brackets is the weighting.

## 4. Conclusions

In this study, different descriptors, different data dividing approaches as well as different statistic methods are used to build QSAR models for inhibitors of Hedgehog signal pathway on 93 cyclopamine derivatives together with their activities against four different cell lines. Our investigation has shown that NCI-466 may serve as the best cell line for testing the activities of inhibitors of Hedgehog signal pathway. Due to the lower qualities of the data, the binary classification method is a much better choice in building QSAR models than regression. Furthermore, for synthesis and medical scientists, our results indicate that demethylation, methylation and hydroxylation at a specific position may highly improve the activity of inhibitors of Hedgehog signal pathway. Demethylation is also found to be a better choice than methylation or hydroxylation for compound modification. Based on these conclusions, demethylation is preferred to methylation or hydroxylation in compound modification and such work is currently being actively pursued in our laboratory.

## Supplementary Materials



## Figures and Tables

**Table 1.** QSAR results derived from the data divided by Diverse Subset (σ indicates difference).

		**BxPC-3**			**NCI-H446**			**SW1990**			**NCI-H157**		
		**General**	**Drug-like**	σ	**General**	**Drug-like**	σ	**General**	**Drug-like**	σ	**General**	**Drug-like**	σ
**PLS**	**R2**	0.552	0.494	−0.058	0.659	0.526	−0.133	0.644	0.585	−0.059	0.527	0.531	0.004
	**Q2**	0.000	0.035	0.035	0.001	0.026	0.025	0.021	0.158	0.137	0.038	0.106	0.068
	**r2**	0.102	0.307	0.205	0.218	0.025	−0.193	0.084	0.193	0.109	0.019	0.118	0.099
**SVR**	**R2**	0.994	0.686	0.308	0.966	0.763	−0.203	0.993	0.808	−0.185	0.988	0.705	−0.283
	**Q2**	0.994	0.000	−0.994	0.962	0.002	−0.96	0.992	0.069	−0.923	0.987	0.001	−0.986
	**r2**	0.000	0.396	0.396	0.088	0.110	0.022	0.025	0.258	0.233	0.023	0.077	0.054
**Bayesian inference**	**At**	0.883	0.917	0.034	1.000	0.967	−0.033	0.900	0.933	0.033	0.967	0.933	−0.034
	**Av**	0.783	0.817	0.034	0.917	0.917	0	0.883	0.783	−0.1	0.867	0.867	0
	**Ap**	0.606	0.576	−0.03	0.758	**0.879**	0.121	0.576	0.667	0.091	0.485	0.636	0.151
**SVM classification**	**At**	1.000	1.000	0	1.000	1.000	0	1.000	1.000	0	1.000	1.000	0
	**Av**	0.550	0.500	−0.05	0.867	0.817	−0.05	0.650	0.533	−0.117	0.633	0.617	−0.016
	**Ap**	0.455	0.636	0.181	0.788	**0.879**	0.091	0.545	0.758	0.213	0.697	0.636	−0.061

**Table 2.** QSAR results derived from the data divided by *Cluster plus Diverse Subset* (σ indicates difference).

		**BxPC-3**			**NCI-H446**			**SW1990**			**NCI-H157**		
		**General**	**Drug-like**	σ	**General**	**Drug-like**	σ	**General**	**Drug-like**	σ	**General**	**Drug-like**	σ
**PLS**	**R2**	0.506	0.474	−0.032	0.593	0.396	−0.197	0.542	0.493	−0.049	0.587	0.542	−0.045
	**Q2**	0.011	0.007	−0.004	0.015	0.019	0.004	0.005	0.002	−0.003	0.006	0.040	0.034
	**r2**	0.178	0.215	0.037	0.055	0.201	0.146	0.000	0.222	0.222	0.087	0.056	−0.031
**SVR**	**R2**	0.997	0.716	−0.281	0.965	0.756	−0.209	0.993	0.839	−0.154	0.987	0.655	−0.332
	**Q2**	0.997	0.021	−0.976	0.962	0.025	−0.937	0.993	0.124	−0.869	0.986	0.019	−0.967
	**r2**	0.008	0.139	0.131	0.029	0.001	−0.028	0.040	0.075	0.035	0.019	0.087	0.068
**Bayesian inference**	**At**	0.967	0.885	−0.082	0.951	0.934	−0.017	0.934	0.918	−0.016	0.984	0.885	−0.099
	**Av**	0.852	0.803	−0.049	0.934	0.918	−0.016	0.852	0.836	−0.016	0.820	0.820	0
	**Ap**	0.656	0.625	−0.031	0.625	**0.906**	0.281	0.625	0.656	0.031	0.625	0.625	0
**SVM classification**	**At**	1.000	0.984	−0.016	1.000	1.000	0	1.000	1.000	0	1.000	0.984	−0.016
	**Av**	0.505	0.475	−0.03	0.803	0.852	0.049	0.590	0.623	0.033	0.656	0.623	−0.033
	**Ap**	0.656	0.719	0.063	0.875	**0.875**	0	0.625	0.719	0.094	0.688	0.719	0.031

## Abbreviations

R2 =: correlation coefficient in self fitting of training data set
Q2 =: correlation coefficient in cross validation fitting of training data set
r2 =: correlation coefficient in fitting of test data set
A =: percentage accuracy of binary model = Total accuracy
A0 =: percentage accuracy of inactive subset
A1 =: percentage accuracy of active subset
At =: A in self fitting of training data set
Av =: A in cross validation fitting of training data set
Ap =: A in fitting of test data set
DLI =: Drug-like Index
PLS =: Partial Least Squares
SVR =: Support Vector Regression
SVM =: Support Vector Machine
ANN =: Artificial Neural Networks
SAReport =: Structure-Activity Report
