# Supplementary material for: Investigations on Inhibitors of Hedgehog Signal Pathway: A Quantitative Structure-Activity Relationship Study
Source: Int J Mol Sci. 2011 May 11;12(5):3018–33. doi: 10.3390/ijms12053018 (PMC3116172; doi:10.3390/ijms12053018)
Supplement: Supplementary file 1 [file ijms-12-03018-s001.doc]

**Figure S1.** Structures of 93 cyclopamine derivatives.

| M-1 | M-2 | M-3 |
| --- | --- | --- |
| M-5 | M-7 | M-10 |
| M-11 | M-13a | M-13b |
| M-14 | M-15 | M-16 |
| M-17 | M-18 | M-19 |

**Figure S1.** *Cont.*

| M-20 | M-21 | M-22 |
| --- | --- | --- |
| M-23 | M-24 | M-25 |
| M-26 | M-27 | M-28 |
| M-29 | M-30 | M-31 |
| M-32 | M-33 | M-35 |

**Figure S1.** *Cont.*

| M-36 | M-37 | M-38 |
| --- | --- | --- |
| M-39 | M-41 | M-42 |
| M-43 | M-44 | M-45 |
| M-46a | M-46b | M-47 |
| M-48 | M-49 | M-50 |

**Figure S1.** *Cont.*

| M-51 | M-52 | M-53 |
| --- | --- | --- |
| M-54 | M-55 | M-56 |
| M-57 | M-58 | M-61 |
| M-62 | M-64 | M-65 |
| M-66 | M-67 | M-70 |

**Figure S1.** *Cont.*

| M-71 | M-72 | M-74 |
| --- | --- | --- |
| M-75 | M-77 | M-78 |
| M-79 | M-80 | M-81 |
| M-82 | M-83 | M-85 |
| M-86 | M-87 | M-88 |

**Figure S1.** *Cont.*

| M-89 | M-90 | M-91a |
| --- | --- | --- |
| M-91b | M-92 | M-93 |
| M-95a | M-95c | M-96a |
| M-96b | M-97 | M-98 |
| M-100 | M-101 | M-102 |

**Figure S1.** *Cont.*

| M-103 | XL-5 | XL-6 |
| --- | --- | --- |

**Table S1.** Activities of 93 cyclopamine derivatives against four different cell lines (NCI-H446, BxPC-3, SW1990 and NCI-H157).

| **Label** | **BxPC-3*** | **NCI-H446** | **SW1990** | **NCI-H157** | **Index** |
| --- | --- | --- | --- | --- | --- |
| **M-1*** | 38.11 | 9.13 | 61.05 | 58.33 | 1 |
| **M-10** | 14.32 | 13.26 | 15.73 | 29.5 | 2 |
| **M-100** | 1434.69 | 1099.3 | 1900.7 | 2029.2 | 3 |
| **M-101** | 28.37 | 14.73 | 32.32 | 33.76 | 4 |
| **M-102** | 1018.57 | 77.69 | 644.47 | 1791.06 | 5 |
| **M-103** | 12.72 | 1.21 | 12.86 | 88.5 | 6 |
| **M-11** | 905.87 | 286.76 | 1341.87 | 1402.3 | 7 |
| **M-13a** | 845.35 | 372.24 | 1159.29 | 1625.81 | 8 |
| **M-13b** | 16.63 | 10.05 | 31.65 | 563.41 | 9 |
| **M-14** | 826.27 | 551.13 | 539.85 | 686.36 | 10 |
| **M-15** | 965.9 | 68.3 | 354.54 | 1241.5 | 11 |
| **M-16** | 55.17 | 40.13 | 77.69 | 603.07 | 12 |
| **M-17** | 498.28 | 30.93 | 52.78 | 1093.06 | 13 |
| **M-18** | 11.22 | 13.69 | 12.58 | 15.04 | 14 |
| **M-19** | 9.18 | 28.17 | 24.6 | 59.54 | 15 |
| **M-2** | 212.96 | 2.26 | 351.79 | 1646.37 | 16 |
| **M-20** | 59.4 | 1784.31 | 22.36 | 1301.29 | 17 |
| **M-21** | 1814.44 | 1090.85 | 1403.6 | 1455.67 | 18 |
| **M-22** | 4.25 | 1902.21 | 31.93 | 22.5 | 19 |
| **M-23** | 728.64 | 1166.94 | 896 | 725.02 | 20 |
| **M-24** | 747.15 | 51.79 | 1360.22 | 994.48 | 21 |
| **M-25** | 39.44 | 25.28 | 11.53 | 1321.13 | 22 |
| **M-26** | 15.72 | 79.76 | 63.08 | 621.53 | 23 |
| **M-27** | 894.26 | 1534.9 | 650.3 | 233.25 | 24 |
| **M-28** | 25.63 | 2141.84 | 51.81 | 9.46 | 25 |
| **M-29** | 10.89 | 35.75 | 32.92 | 16.02 | 26 |
| **M-3** | 16.05 | 5.35 | 10.1 | 960.94 | 27 |
| **M-30** | 7.9 | 1747.95 | 336.92 | 25 | 28 |
| **M-31** | 591.06 | 288.26 | 665.46 | 1486.62 | 29 |
| **M-32** | 11.38 | 56.69 | 182.8 | 29.69 | 30 |
| **M-33** | 1313.36 | 686.17 | 660.87 | 1412.56 | 31 |
| **M-35** | 12.78 | 88.7 | 25.95 | 15.1 | 32 |
| **M-36** | 13.39 | 46.59 | 375.79 | 1306.32 | 33 |

**Table S1. *Cont.***

| **Label** | **BxPC-3*** | **NCI-H446** | **SW1990** | **NCI-H157** | **Index** |
| --- | --- | --- | --- | --- | --- |
| **M-37** | 15.21 | 45.26 | 672.62 | 235.23 | 34 |
| **M-38** | 9.74 | 11.92 | 36.03 | 21.05 | 35 |
| **M-39** | 87.36 | 957.7 | 236.65 | 2010.28 | 36 |
| **M-41** | 1.07 | 11.12 | 15.72 | 25.84 | 37 |
| **M-42** | 19.38 | 14.83 | 29.84 | 33.18 | 38 |
| **M-43** | 17.3 | 9.71 | 16.59 | 31.62 | 39 |
| **M-44** | 7.78 | 5.61 | 7.12 | 7.05 | 40 |
| **M-45** | 11.53 | 12.78 | 13.76 | 31.62 | 41 |
| **M-46a** | 1259.74 | 439.91 | 820.56 | 900.56 | 42 |
| **M-46b** | 15.59 | 11.57 | 15.99 | 1648.94 | 43 |
| **M-47** | 668.6 | 222.65 | 761.74 | 1204.56 | 44 |
| **M-48** | 11.16 | 14.69 | 17.19 | 19.3 | 45 |
| **M-49** | 15.73 | 6.86 | 16.45 | 854.36 | 46 |
| **M-5** | 34.85 | 16.87 | 41.17 | 96.28 | 47 |
| **M-50** | 12.72 | 1.21 | 12.86 | 1572.6 | 48 |
| **M-51** | 20.71 | 59.92 | 26.29 | 1367.04 | 49 |
| **M-52** | 39.86 | 13.36 | 30.75 | 23.12 | 50 |
| **M-53** | 15.05 | 213.76 | 1008.95 | 1077.34 | 51 |
| **M-54** | 1216.62 | 725.49 | 881.17 | 2307.53 | 52 |
| **M-55** | 18.36 | 41.09 | 58.67 | 75.6 | 53 |
| **M-56** | 12.58 | 32.91 | 24.82 | 27.51 | 54 |
| **M-57** | 13.34 | 35.87 | 32.65 | 34.41 | 55 |
| **M-58** | 31.83 | 21.19 | 38.23 | 34 | 56 |
| **M-61** | 31.62 | 17.44 | 30.4 | 2147.99 | 57 |
| **M-62** | 15.45 | 19.58 | 14.24 | 11.98 | 58 |
| **M-64** | 1190.58 | 232.76 | 408.15 | 1676.46 | 59 |
| **M-65** | 184.61 | 76.55 | 1137.59 | 1711.19 | 60 |
| **M-66** | 16.79 | 21.24 | 15.86 | 31.62 | 61 |
| **M-67** | 2.78 | 15.04 | 11.7 | 32.7 | 62 |
| **M-7** | 1260.91 | 1317.19 | 1662.49 | 1753.99 | 63 |
| **M-70** | 572.48 | 1511.92 | 1033.01 | 1848.54 | 64 |
| **M-71** | 1047.45 | 1373.27 | 773.17 | 1646.37 | 65 |
| **M-72** | 740.53 | 431.5 | 615.36 | 1507.51 | 66 |
| **M-74** | 316.9 | 1117.73 | 1445.28 | 1034.09 | 67 |
| **M-75** | 919.6 | 161.89 | 389.19 | 675.72 | 68 |
| **M-77** | 1318.61 | 447.02 | 485.79 | 622.03 | 69 |
| **M-78** | 246.28 | 301.45 | 1446.44 | 1468.58 | 70 |
| **M-79** | 1226.67 | 71.9 | 840.44 | 3019.98 | 71 |
| **M-80** | 17.79 | 31.62 | 45.68 | 61.62 | 72 |
| **M-81** | 1708.75 | 969.9 | 1253.88 | 1330.56 | 73 |
| **M-82** | 1302.13 | 744.51 | 693.72 | 1212.76 | 74 |
| **M-83** | 12.05 | 24.75 | 13.94 | 31.62 | 75 |
| **M-85** | 20.87 | 18.2 | 17.69 | 13.56 | 76 |
| **M-86** | 212.34 | 53.18 | 468.64 | 1523.4 | 77 |
| **M-87** | 985.84 | 65.79 | 728.18 | 1239.4 | 78 |

**Table S1.** *Cont.*

| **Label** | **BxPC-3*** | **NCI-H446** | **SW1990** | **NCI-H157** | **Index** |
| --- | --- | --- | --- | --- | --- |
| **M-88** | 17.79 | 10.51 | 32.81 | 244.29 | 79 |
| **M-89** | 33.01 | 28.54 | 32.25 | 582.5 | 80 |
| **M-90** | 197.27 | 171.16 | 118.36 | 567.66 | 81 |
| **M-91a** | 59.19 | 19.02 | 80.53 | 637.29 | 82 |
| **M-91b** | 6.35 | 0.566 | 3.58 | 31.64 | 83 |
| **M-92** | 813.92 | 27.9 | 635.3 | 1735.94 | 84 |
| **M-93** | 256.77 | 281.83 | 1137.19 | 1215.2 | 85 |
| **M-95a** | 29.43 | 40.01 | 45.94 | 20.03 | 86 |
| **M-95c** | 831.78 | 655.56 | 575.74 | 1365.68 | 87 |
| **M-96a** | 604.23 | 66.46 | 663.08 | 1936.19 | 88 |
| **M-96b** | 1127.33 | 589.77 | 781.87 | 1178.1 | 89 |
| **M-97** | 11.23 | 10 | 11.98 | 31.62 | 90 |
| **M-98** | 1815.6 | 301.71 | 1141.77 | 1322.36 | 91 |
| **XL-5** | 20.69 | 10.29 | 15.25 | 55.26 | 92 |
| **XL-6** | 1565.14 | 1252.55 | 1170.99 | 1820.19 | 93 |

- IC_50_(μg/mL)

**Figure S2.** SAReport.

| **Precursor** | | **Structure** | **BxPC-3** | **NCI-H446** | **SW1990** | **NCI-H157** |
| --- | --- | --- | --- | --- | --- | --- |
| **#1** | **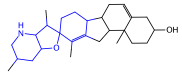** | **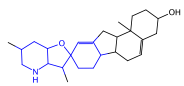** | **+0.00%**  **( X 0.0 )** | **+90.43%**  **( X 47.6 )** | **+0.00%**  **( X 0.0 )** | **+0.00%**  **( X 0.0 )** |
| **#10** | **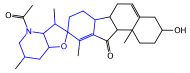** | **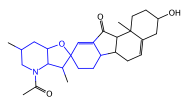** | **+0.00%**  **( X 0.0 )** | **+90.30%**  **( X 46.8 )** | **+0.00%**  **( X 0.0 )** | **+0.00%**  **( X 0.0 )** |
| **#11** | **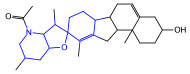** | **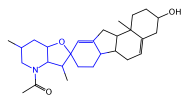** | **+0.00%**  **( X 0.0 )** | **+90.28%**  **( X 46.7 )** | **+0.00%**  **( X 0.0 )** | **+0.00%**  **( X 0.0 )** |
| **#20** | **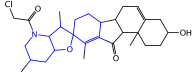** | **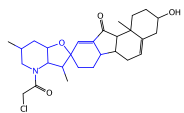** | **+0.00%**  **( X 0.0 )** | **+90.28%**  **( X 46.7 )** | **+0.00%**  **( X 0.0 )** | **+0.00%**  **( X 0.0 )** |
| **#38** | **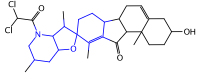** | **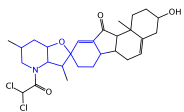** | **+0.00%**  **( X 0.0 )** | **+90.28%**  **( X 46.7 )** | **+0.00%**  **( X 0.0 )** | **+0.00%**  **( X 0.0 )** |

***Figure S2.*** *Cont.*

| **Precursor** | | **Structure** | **BxPC-3** | **NCI-H446** | **SW1990** | **NCI-H157** |
| --- | --- | --- | --- | --- | --- | --- |
| **#22** | **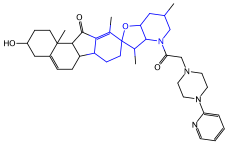** | **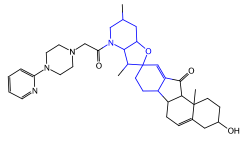** | **+0.00%**  **( X 0.0 )** | **+90.27%**  **( X 46.6 )** | **+0.00%**  **( X 0.0 )** | **+0.00%**  **( X 0.0 )** |
| **#23** | **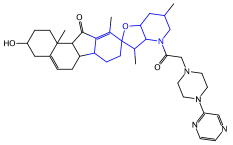** | **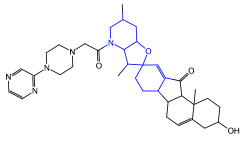** | **+0.00%**  **( X 0.0 )** | **+90.27%**  **( X 46.6 )** | **+0.00%**  **( X 0.0 )** | **+0.00%**  **( X 0.0 )** |
| **#24** | **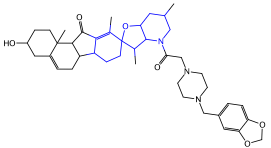** | **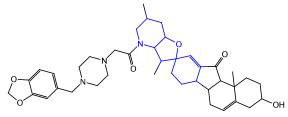** | **+0.00%**  **( X 0.0 )** | **+90.27%**  **( X 46.6 )** | **+0.00%**  **( X 0.0 )** | **+0.00%**  **( X 0.0 )** |
| **#49** | **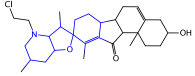** | **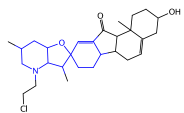** | **+0.00%**  **( X 0.0 )** | **+90.27%**  **( X 46.6 )** | **+0.00%**  **( X 0.0 )** | **+0.00%**  **( X 0.0 )** |
| **#50** | 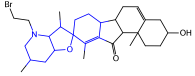 | 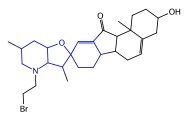 | **+0.00%**  **( X 0.0 )** | **+90.27%**  **( X 46.6 )** | **+0.00%**  **( X 0.0 )** | **+0.00%**  **( X 0.0 )** |
| **#52** | 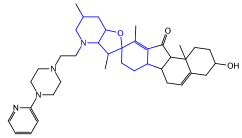 | 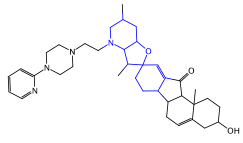 | **+0.00%**  **( X 0.0 )** | **+90.27%**  **( X 46.6 )** | **+0.00%**  **( X 0.0 )** | **+0.00%**  **( X 0.0 )** |
| **#53** | **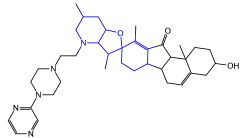** | **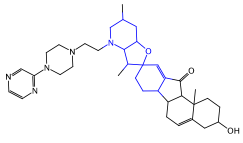** | **+0.00%**  **( X 0.0 )** | **+90.27%**  **( X 46.6 )** | **+0.00%**  **( X 0.0 )** | **+0.00%**  **( X 0.0 )** |
| **#54** | **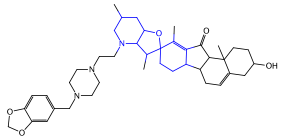** | **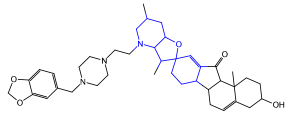** | **+0.00%**  **( X 0.0 )** | **+90.27%**  **( X 46.6 )** | **+0.00%**  **( X 0.0 )** | **+0.00%**  **( X 0.0 )** |

***Figure S2.*** *Cont.*

| **Precursor** | | **Structure** | **BxPC-3** | **NCI-H446** | **SW1990** | **NCI-H157** |
| --- | --- | --- | --- | --- | --- | --- |
| **#56** | **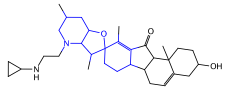** | **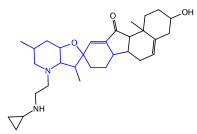** | **+0.00%**  **( X 0.0 )** | **+90.27%**  **( X 46.6 )** | **+0.00%**  **( X 0.0 )** | **+0.00%**  **( X 0.0 )** |
| **#70** | **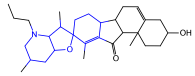** | **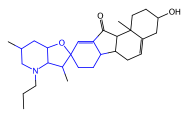** | **+0.00%**  **( X 0.0 )** | **+90.27%**  **( X 46.6 )** | **+0.00%**  **( X 0.0 )** | **+0.00%**  **( X 0.0 )** |
| **#74** | **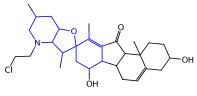** | **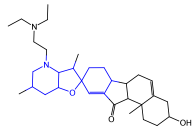** | **+0.00%**  **( X 0.0 )** | **+90.27%**  **( X 46.6 )** | **+0.00%**  **( X 0.0 )** | **+0.00%**  **( X 0.0 )** |
| **#76** | **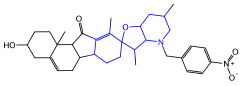** | **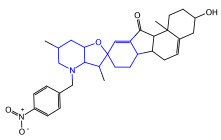** | **+0.00%**  **( X 0.0 )** | **+90.27%**  **( X 46.6 )** | **+0.00%**  **( X 0.0 )** | **+0.00%**  **( X 0.0 )** |
| **#84** | **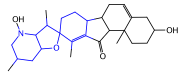** | **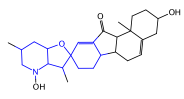** | **+0.00%**  **( X 0.0 )** | **+90.27%**  **( X 46.6 )** | **+0.00%**  **( X 0.0 )** | **+0.00%**  **( X 0.0 )** |
| **#21** | **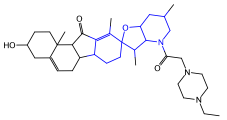** | **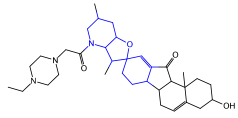** | **+0.00%**  **( X 0.0 )** | **+90.26%**  **( X 46.6 )** | **+0.00%**  **( X 0.0 )** | **+0.00%**  **( X 0.0 )** |
| **#25** | **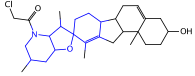** | **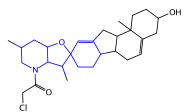** | **+0.00%**  **( X 0.0 )** | **+90.26%**  **( X 46.6 )** | **+0.00%**  **( X 0.0 )** | **+0.00%**  **( X 0.0 )** |
| **#39** | **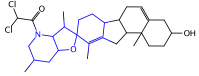** | **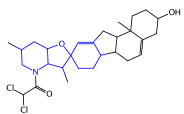** | **+0.00%**  **( X 0.0 )** | **+90.26%**  **( X 46.6 )** | **+0.00%**  **( X 0.0 )** | **+0.00%**  **( X 0.0 )** |
| **#42** | **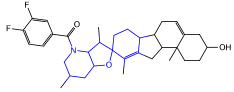** | **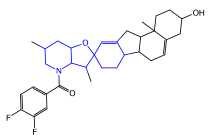** | **+0.00%**  **( X 0.0 )** | **+90.26%**  **( X 46.6 )** | **+0.00%**  **( X 0.0 )** | **+0.00%**  **( X 0.0 )** |
| **#51** | **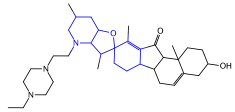** | **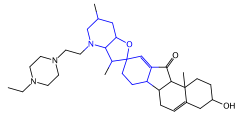** | **+0.00%**  **( X 0.0 )** | **+90.26%**  **( X 46.6 )** | **+0.00%**  **( X 0.0 )** | **+0.00%**  **( X 0.0 )** |

***Figure S2.*** *Cont.*

| **Precursor** | | **Structure** | **BxPC-3** | **NCI-H446** | **SW1990** | **NCI-H157** |
| --- | --- | --- | --- | --- | --- | --- |
| **#55** | **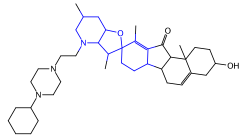** | **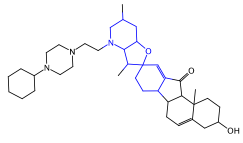** | **+0.00%**  **( X 0.0 )** | **+90.26%**  **( X 46.6 )** | **+0.00%**  **( X 0.0 )** | **+0.00%**  **( X 0.0 )** |
| **#71** | **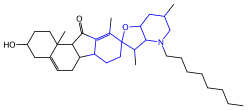** | **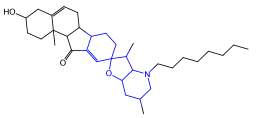** | **+0.00%**  **( X 0.0 )** | **+90.26%**  **( X 46.6 )** | **+0.00%**  **( X 0.0 )** | **+0.00%**  **( X 0.0 )** |
| **#79** | **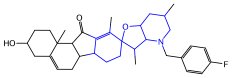** | **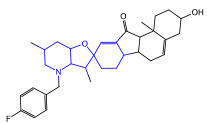** | **+0.00%**  **( X 0.0 )** | **+90.26%**  **( X 46.6 )** | **+0.00%**  **( X 0.0 )** | **+0.00%**  **( X 0.0 )** |
| **#28** | **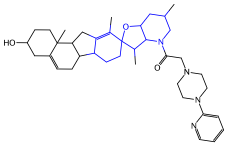** | **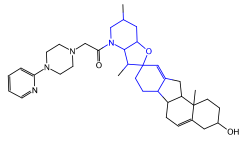** | **+0.00%**  **( X 0.0 )** | **+90.25%**  **( X 46.5 )** | **+0.00%**  **( X 0.0 )** | **+0.00%**  **( X 0.0 )** |
| **#29** | **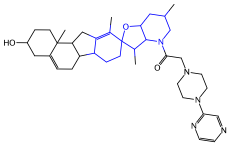** | **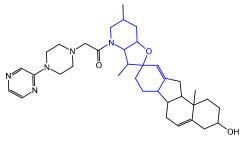** | **+0.00%**  **( X 0.0 )** | **+90.25%**  **( X 46.5 )** | **+0.00%**  **( X 0.0 )** | **+0.00%**  **( X 0.0 )** |
| **#30** | **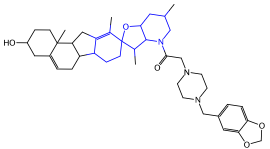** | **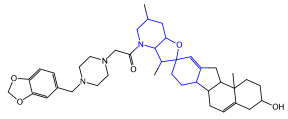** | **+0.00%**  **( X 0.0 )** | **+90.25%**  **( X 46.5 )** | **+0.00%**  **( X 0.0 )** | **+0.00%**  **( X 0.0 )** |
| **#57** | **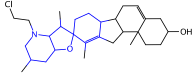** | **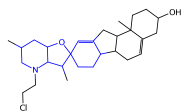** | **+0.00%**  **( X 0.0 )** | **+90.25%**  **( X 46.5 )** | **+0.00%**  **( X 0.0 )** | **+0.00%**  **( X 0.0 )** |
| **#58** | **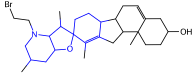** | **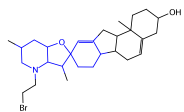** | **+0.00%**  **( X 0.0 )** | **+90.25%**  **( X 46.5 )** | **+0.00%**  **( X 0.0 )** | **+0.00%**  **( X 0.0 )** |
| **#60** | **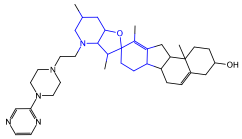** | **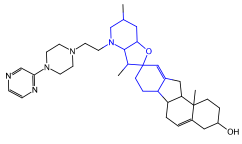** | **+0.00%**  **( X 0.0 )** | **+90.25%**  **( X 46.5 )** | **+0.00%**  **( X 0.0 )** | **+0.00%**  **( X 0.0 )** |
| **#61** | **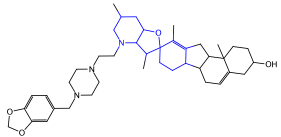** | **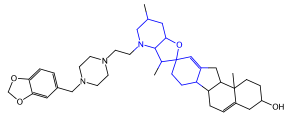** | **+0.00%**  **( X 0.0 )** | **+90.25%**  **( X 46.5 )** | **+0.00%**  **( X 0.0 )** | **+0.00%**  **( X 0.0 )** |

***Figure S2.*** *Cont.*

| **Precursor** | | **Structure** | **BxPC-3** | **NCI-H446** | **SW1990** | **NCI-H157** |
| --- | --- | --- | --- | --- | --- | --- |
| **#62** | **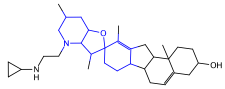** | **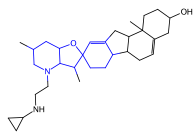** | **+0.00%**  **( X 0.0 )** | **+90.25%**  **( X 46.5 )** | **+0.00%**  **( X 0.0 )** | **+0.00%**  **( X 0.0 )** |
| **#72** | **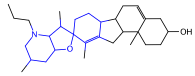** | **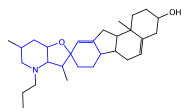** | **+0.00%**  **( X 0.0 )** | **+90.25%**  **( X 46.5 )** | **+0.00%**  **( X 0.0 )** | **+0.00%**  **( X 0.0 )** |
| **#75** | **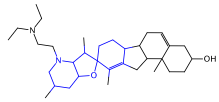** | **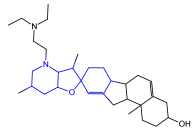** | **+0.00%**  **( X 0.0 )** | **+90.25%**  **( X 46.5 )** | **+0.00%**  **( X 0.0 )** | **+0.00%**  **( X 0.0 )** |
| **#77** | **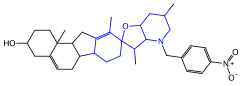** | **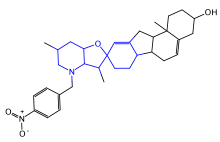** | **+0.00%**  **( X 0.0 )** | **+90.25%**  **( X 46.5 )** | **+0.00%**  **( X 0.0 )** | **+0.00%**  **( X 0.0 )** |
| **#31** | **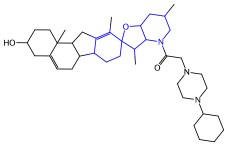** | **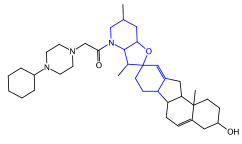** | **+0.00%**  **( X 0.0 )** | **+90.24%**  **( X 46.4 )** | **+0.00%**  **( X 0.0 )** | **+0.00%**  **( X 0.0 )** |
| **#73** | **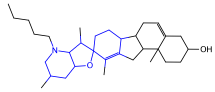** | **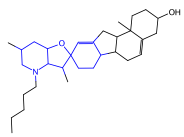** | **+0.00%**  **( X 0.0 )** | **+90.24%**  **( X 46.4 )** | **+0.00%**  **( X 0.0 )** | **+0.00%**  **( X 0.0 )** |
| **#93** | **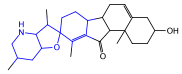** | **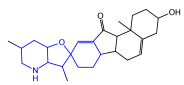** | **+0.00%**  **( X 0.0 )** | **+90.22%**  **( X 46.4 )** | **+0.00%**  **( X 0.0 )** | **+0.00%**  **( X 0.0 )** |
| **#85** | **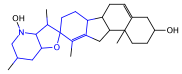** | **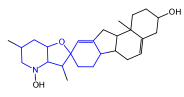** | **+0.00%**  **( X 0.0 )** | **+90.024%**  **( X 45.2 )** | **+0.00%**  **( X 0.0 )** | **+0.00%**  **( X 0.0 )** |
| **#47** | **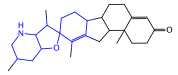** | **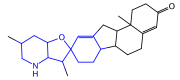** | **+0.00%**  **( X 0.0 )** | **+89.99%**  **( X 45.1)** | **+0.00%**  **( X 0.0 )** | **+0.00%**  **( X 0.0 )** |
| **#88** | **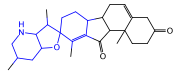** | **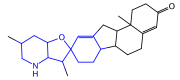** | **+0.00%**  **( X 0.0 )** | **+89.99%**  **( X 45.1)** | **+0.00%**  **( X 0.0 )** | **+0.00%**  **( X 0.0 )** |
| **#3** | **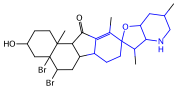** | **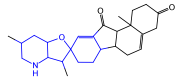** | **+0.00%**  **( X 0.0 )** | **+89.98%**  **( X 45.0)** | **+0.00%**  **( X 0.0 )** | **+0.00%**  **( X 0.0 )** |

***Figure S2.*** *Cont.*

| **Precursor** | | **Structure** | **BxPC-3** | **NCI-H446** | **SW1990** | **NCI-H157** |
| --- | --- | --- | --- | --- | --- | --- |
| **#7** | **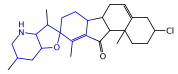** | **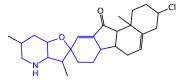** | **+0.00%**  **( X 0.0 )** | **+89.98%**  **( X 45.0)** | **+0.00%**  **( X 0.0 )** | **+0.00%**  **( X 0.0 )** |
| **#13** | **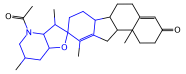** | **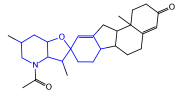** | **+0.00%**  **( X 0.0 )** | **+89.82%**  **( X 44.2)** | **+0.00%**  **( X 0.0 )** | **+0.00%**  **( X 0.0 )** |
| **#91** | **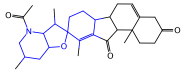** | **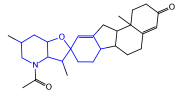** | **+0.00%**  **( X 0.0 )** | **+89.82%**  **( X 44.2)** | **+0.00%**  **( X 0.0 )** | **+0.00%**  **( X 0.0 )** |
| **#89** | **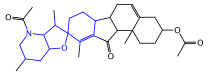** | **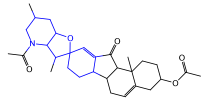** | **+0.00%**  **( X 0.0 )** | **+89.81%**  **( X 44.1)** | **+0.00%**  **( X 0.0 )** | **+0.00%**  **( X 0.0 )** |
| **#41** | **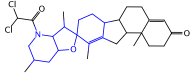** | **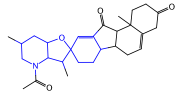** | **+0.00%**  **( X 0.0 )** | **+89.80%**  **( X 44.1)** | **+0.00%**  **( X 0.0 )** | **+0.00%**  **( X 0.0 )** |
| **#44** | **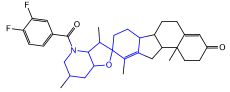** | **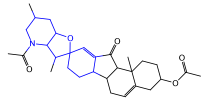** | **+0.00%**  **( X 0.0 )** | **+89.80%**  **( X 44.1)** | **+0.00%**  **( X 0.0 )** | **+0.00%**  **( X 0.0 )** |
| **#43** |  |  | **+0.00%**  **( X 0.0 )** | **+89.79%**  **( X 44.0)** | **+0.00%**  **( X 0.0 )** | **+0.00%**  **( X 0.0 )** |
| **#27** |  |  | **+0.00%**  **( X 0.0 )** | **+78.41%**  **( X 21.2)** | **+0.00%**  **( X 0.0 )** | **+0.00%**  **( X 0.0 )** |
| **#12** |  |  | **+0.00%**  **( X 0.0 )** | **+78.36%**  **( X 21.1)** | **+0.00%**  **( X 0.0 )** | **+0.00%**  **( X 0.0 )** |
| **#32** |  |  | **+0.00%**  **( X 0.0 )** | **+78.36%**  **( X 21.1)** | **+0.00%**  **( X 0.0 )** | **+0.00%**  **( X 0.0 )** |
| **#33** |  |  | **+0.00%**  **( X 0.0 )** | **+78.36%**  **( X 21.1)** | **+0.00%**  **( X 0.0 )** | **+0.00%**  **( X 0.0 )** |

***Figure S2.*** *Cont.*

| **Precursor** | | **Structure** | **BxPC-3** | **NCI-H446** | **SW1990** | **NCI-H157** |
| --- | --- | --- | --- | --- | --- | --- |
| **#34** |  |  | **+0.00%**  **( X 0.0 )** | **+78.36%**  **( X 21.1)** | **+0.00%**  **( X 0.0 )** | **+0.00%**  **( X 0.0 )** |
| **#35** |  |  | **+0.00%**  **( X 0.0 )** | **+78.36%**  **( X 21.1)** | **+0.00%**  **( X 0.0 )** | **+0.00%**  **( X 0.0 )** |
| **#36** |  |  | **+0.00%**  **( X 0.0 )** | **+78.36%**  **( X 21.1)** | **+0.00%**  **( X 0.0 )** | **+0.00%**  **( X 0.0 )** |
| **#40** |  |  | **+0.00%**  **( X 0.0 )** | **+78.36%**  **( X 21.1)** | **+0.00%**  **( X 0.0 )** | **+0.00%**  **( X 0.0 )** |
| **#69** |  |  | **+0.00%**  **( X 0.0 )** | **+78.36%**  **( X 21.1)** | **+0.00%**  **( X 0.0 )** | **+0.00%**  **( X 0.0 )** |
| **#78** |  |  | **+0.00%**  **( X 0.0 )** | **+78.36%**  **( X 21.1)** | **+0.00%**  **( X 0.0 )** | **+0.00%**  **( X 0.0 )** |
| **#81** |  |  | **+0.00%**  **( X 0.0 )** | **+78.36%**  **( X 21.1)** | **+0.00%**  **( X 0.0 )** | **+0.00%**  **( X 0.0 )** |

***Figure S2.*** *Cont.*

| **Precursor** | | **Structure** | **BxPC-3** | **NCI-H446** | **SW1990** | **NCI-H157** |
| --- | --- | --- | --- | --- | --- | --- |
| **#86** |  |  | **+0.00%**  **( X 0.0 )** | **+73.72%**  **( X 16.7)** | **+0.00%**  **( X 0.0 )** | **+0.00%**  **( X 0.0 )** |
| **#90** |  |  | **+0.00%**  **( X 0.0 )** | **+73.64%**  **( X 16.6)** | **+0.00%**  **( X 0.0 )** | **+0.00%**  **( X 0.0 )** |
| **#1** |  |  | **+0.00%**  **( X 0.0 )** | **+8.88%**  **( X 48.1)** | **+0.00%**  **( X 0.0 )** | **+0.00%**  **( X 0.0 )** |
| **#10** |  |  | **+0.00%**  **( X 0.0 )** | **+8.99%**  **( X 47.3)** | **+0.00%**  **( X 0.0 )** | **+0.00%**  **( X 0.0 )** |
| **#11** |  |  | **+0.00%**  **( X 0.0 )** | **+9.01%**  **( X 47.2)** | **+0.00%**  **( X 0.0 )** | **+0.00%**  **( X 0.0 )** |
| **#20** |  |  | **+0.00%**  **( X 0.0 )** | **+9.01%**  **( X 47.2)** | **+0.00%**  **( X 0.0 )** | **+0.00%**  **( X 0.0 )** |
| **#38** |  |  | **+0.00%**  **( X 0.0 )** | **+9.01%**  **( X 47.2)** | **+0.00%**  **( X 0.0 )** | **+0.00%**  **( X 0.0 )** |
| **#22** |  |  | **+0.00%**  **( X 0.0 )** | **+9.02%**  **( X 47.1)** | **+0.00%**  **( X 0.0 )** | **+0.00%**  **( X 0.0 )** |
| **#23** |  |  | **+0.00%**  **( X 0.0 )** | **+9.02%**  **( X 47.1)** | **+0.00%**  **( X 0.0 )** | **+0.00%**  **( X 0.0 )** |
| **#24** |  |  | **+0.00%**  **( X 0.0 )** | **+9.02%**  **( X 47.1)** | **+0.00%**  **( X 0.0 )** | **+0.00%**  **( X 0.0 )** |
| **#49** |  |  | **+0.00%**  **( X 0.0 )** | **+9.02%**  **( X 47.1)** | **+0.00%**  **( X 0.0 )** | **+0.00%**  **( X 0.0 )** |

***Figure S2.*** *Cont.*

| **Precursor** | | **Structure** | **BxPC-3** | **NCI-H446** | **SW1990** | **NCI-H157** |
| --- | --- | --- | --- | --- | --- | --- |
| **#50** |  |  | **+0.00%**  **( X 0.0 )** | **+9.02%**  **( X 47.1)** | **+0.00%**  **( X 0.0 )** | **+0.00%**  **( X 0.0 )** |
| **#52** |  |  | **+0.00%**  **( X 0.0 )** | **+9.02%**  **( X 47.1)** | **+0.00%**  **( X 0.0 )** | **+0.00%**  **( X 0.0 )** |
| **#53** |  |  | **+0.00%**  **( X 0.0 )** | **+9.02%**  **( X 47.1)** | **+0.00%**  **( X 0.0 )** | **+0.00%**  **( X 0.0 )** |
| **#54** |  |  | **+0.00%**  **( X 0.0 )** | **+9.02%**  **( X 47.1)** | **+0.00%**  **( X 0.0 )** | **+0.00%**  **( X 0.0 )** |
| **#56** |  |  | **+0.00%**  **( X 0.0 )** | **+9.02%**  **( X 47.1)** | **+0.00%**  **( X 0.0 )** | **+0.00%**  **( X 0.0 )** |
| **#70** |  |  | **+0.00%**  **( X 0.0 )** | **+9.02%**  **( X 47.1)** | **+0.00%**  **( X 0.0 )** | **+0.00%**  **( X 0.0 )** |
| **#74** |  |  | **+0.00%**  **( X 0.0 )** | **+9.02%**  **( X 47.1)** | **+0.00%**  **( X 0.0 )** | **+0.00%**  **( X 0.0 )** |
| **#76** |  |  | **+0.00%**  **( X 0.0 )** | **+9.02%**  **( X 47.1)** | **+0.00%**  **( X 0.0 )** | **+0.00%**  **( X 0.0 )** |
| **#84** |  |  | **+0.00%**  **( X 0.0 )** | **+9.02%**  **( X 47.1)** | **+0.00%**  **( X 0.0 )** | **+0.00%**  **( X 0.0 )** |
| **#21** |  |  | **+0.00%**  **( X 0.0 )** | **+9.03%**  **( X 47.1)** | **+0.00%**  **( X 0.0 )** | **+0.00%**  **( X 0.0 )** |
| **#25** |  |  | **+0.00%**  **( X 0.0 )** | **+9.03%**  **( X 47.1)** | **+0.00%**  **( X 0.0 )** | **+0.00%**  **( X 0.0 )** |

***Figure S2.*** *Cont.*

| **Precursor** | | **Structure** | **BxPC-3** | **NCI-H446** | **SW1990** | **NCI-H157** |
| --- | --- | --- | --- | --- | --- | --- |
| **#39** |  |  | **+0.00%**  **( X 0.0 )** | **+9.03%**  **( X 47.1)** | **+0.00%**  **( X 0.0 )** | **+0.00%**  **( X 0.0 )** |
| **#42** |  |  | **+0.00%**  **( X 0.0 )** | **+9.03%**  **( X 47.1)** | **+0.00%**  **( X 0.0 )** | **+0.00%**  **( X 0.0 )** |
| **#51** |  |  | **+0.00%**  **( X 0.0 )** | **+9.03%**  **( X 47.1)** | **+0.00%**  **( X 0.0 )** | **+0.00%**  **( X 0.0 )** |
| **#55** |  |  | **+0.00%**  **( X 0.0 )** | **+9.03%**  **( X 47.1)** | **+0.00%**  **( X 0.0 )** | **+0.00%**  **( X 0.0 )** |
| **#71** |  |  | **+0.00%**  **( X 0.0 )** | **+9.03%**  **( X 47.1)** | **+0.00%**  **( X 0.0 )** | **+0.00%**  **( X 0.0 )** |
| **#79** |  |  | **+0.00%**  **( X 0.0 )** | **+9.03%**  **( X 47.1)** | **+0.00%**  **( X 0.0 )** | **+0.00%**  **( X 0.0 )** |
| **#28** |  |  | **+0.00%**  **( X 0.0 )** | **+9.04%**  **( X 47.0)** | **+0.00%**  **( X 0.0 )** | **+0.00%**  **( X 0.0 )** |
| **#29** |  |  | **+0.00%**  **( X 0.0 )** | **+9.04%**  **( X 47.0)** | **+0.00%**  **( X 0.0 )** | **+0.00%**  **( X 0.0 )** |
| **#30** |  |  | **+0.00%**  **( X 0.0 )** | **+9.04%**  **( X 47.0)** | **+0.00%**  **( X 0.0 )** | **+0.00%**  **( X 0.0 )** |
| **#57** |  |  | **+0.00%**  **( X 0.0 )** | **+9.04%**  **( X 47.0)** | **+0.00%**  **( X 0.0 )** | **+0.00%**  **( X 0.0 )** |
| **#58** |  |  | **+0.00%**  **( X 0.0 )** | **+9.04%**  **( X 47.0)** | **+0.00%**  **( X 0.0 )** | **+0.00%**  **( X 0.0 )** |

***Figure S2.*** *Cont.*

| **Precursor** | | **Structure** | **BxPC-3** | **NCI-H446** | **SW1990** | **NCI-H157** |
| --- | --- | --- | --- | --- | --- | --- |
| **#59** |  |  | **+0.00%**  **( X 0.0 )** | **+9.04%**  **( X 47.0)** | **+0.00%**  **( X 0.0 )** | **+0.00%**  **( X 0.0 )** |
| **#60** |  |  | **+0.00%**  **( X 0.0 )** | **+9.04%**  **( X 47.0)** | **+0.00%**  **( X 0.0 )** | **+0.00%**  **( X 0.0 )** |
| **#61** |  |  | **+0.00%**  **( X 0.0 )** | **+9.04%**  **( X 47.0)** | **+0.00%**  **( X 0.0 )** | **+0.00%**  **( X 0.0 )** |
| **#62** |  |  | **+0.00%**  **( X 0.0 )** | **+9.04%**  **( X 47.0)** | **+0.00%**  **( X 0.0 )** | **+0.00%**  **( X 0.0 )** |

**Table S2.** The general descriptors include: SlogP_VSAk (10) intended to capture hydrophobic and hydrophilic effects either in the receptor or on the way to the receptor; SMR_VSAk (8) intended to capture polarizability; and PEOE_VSAk (14) intended to capture direct electrostatic interactions.

| **Code** | **Description** |
| --- | --- |
| SlogP_VSA0 | Sum of vi such that Li <= −0.4. |
| SlogP_VSA1 | Sum of vi such that Li is in (−0.4, −0.2]. |
| SlogP_VSA2 | Sum of vi such that Li is in (−0.2,0]. |
| SlogP_VSA3 | Sum of vi such that Li is in (0,0.1]. |
| SlogP_VSA4 | Sum of vi such that Li is in (0.1,0.15]. |
| SlogP_VSA5 | Sum of vi such that Li is in (0.15,0.20]. |
| SlogP_VSA6 | Sum of vi such that Li is in (0.20,0.25]. |
| SlogP_VSA7 | Sum of vi such that Li is in (0.25,0.30]. |
| SlogP_VSA8 | Sum of vi such that Li is in (0.30,0.40]. |
| SlogP_VSA9 | Sum of vi such that Li > 0.40. |
| SMR_VSA0 | Sum of vi such that Ri is in [0,0.11]. |
| SMR_VSA1 | Sum of vi such that Ri is in (0.11,0.26]. |

***Table S2.*** *Cont.*

| **Code** | **Description** |
| --- | --- |
| SMR_VSA2 | Sum of vi such that Ri is in (0.26,0.35]. |
| SMR_VSA3 | Sum of vi such that Ri is in (0.35,0.39]. |
| SMR_VSA4 | Sum of vi such that Ri is in (0.39,0.44]. |
| SMR_VSA5 | Sum of vi such that Ri is in (0.44,0.485]. |
| SMR_VSA6 | Sum of vi such that Ri is in (0.485,0.56]. |
| SMR_VSA7 | Sum of vi such that Ri > 0.56. |
| PEOE_VSA+6 | Sum of vi where qi is greater than 0.3. |
| PEOE_VSA+5 | Sum of vi where qi is in the range [0.25,0.30). |
| PEOE_VSA+4 | Sum of vi where qi is in the range [0.20,0.25). |
| PEOE_VSA+3 | Sum of vi where qi is in the range [0.15,0.20). |
| PEOE_VSA+2 | Sum of vi where qi is in the range [0.10,0.15). |
| PEOE_VSA+1 | Sum of vi where qi is in the range [0.05,0.10). |
| PEOE_VSA+0 | Sum of vi where qi is in the range [0.00,0.05). |
| PEOE_VSA−0 | Sum of vi where qi is in the range [−0.05,0.00). |
| PEOE_VSA−1 | Sum of vi where qi is in the range [−0.10,−0.05). |
| PEOE_VSA−2 | Sum of vi where qi is in the range [−0.15,−0.10). |
| PEOE_VSA−3 | Sum of vi where qi is in the range [−0.20,−0.15). |
| PEOE_VSA−4 | Sum of vi where qi is in the range [−0.25,−0.20). |
| PEOE_VSA−5 | Sum of vi where qi is in the range [−0.30,−0.25). |
| PEOE_VSA−6 | Sum of vi where qi is less than −0.30. |

The Subdivided Surface Areas are descriptors(SlogP_VSAk and SMR_VSAk) based on an approximate accessible van der Waals surface area (in Å2) calculation for each atom, vi along with some other atomic property, pi. The vi are calculated using a connection table approximation. Each descriptor in a series is defined to be the sum of the vi over all atoms i such that pi is in a specified range (a,b). In the descriptions to follow, Li denotes the contribution to logP(o/w) for atom i as calculated in the SlogP descriptor. Ri denotes the contribution to Molar Refractivity for atom i as calculated in the SMR descriptor. The ranges were determined by percentile subdivision over a large collection of compounds.

For Partial Charge Descriptors(PEOE_VSAk), let qi denote the partial charge of atom i as defined above, and let vi be the van der Waals surface area (Å2) of atom i (as calculated by a connection table approximation).

**Table S3.** The Drug-Like Index descriptors include.

| DLI(01) | '# of non-H', | DLI(02) | 'total SSSR size', |
| --- | --- | --- | --- |
| DLI(03) | 'degree of cyclization', | DLI(04) | '# of rotatable bonds', |
| DLI(05) | '# of non-H polar bonds', | DLI(06) | '# of carbons in cap fragments', |
| DLI(07) | '# of N with # of H > 0', | DLI(08) | '# of hydroxyl groups', |
| DLI(09) | '# of H-bond donors', | DLI(10) | '# of H-bond acceptors', |
| DLI(11) | '# of N and O atoms', | DLI(12) | '# of 2-degree acyclic atoms', |
| DLI(13) | '# of 2-degree cyclic atoms', | DLI(14) | '# of 3-degree acyclic atoms', |
| DLI(15) | '# of 3-degree cyclic atoms', | DLI(16) | '# of 1-level bonding patterns', |
| DLI(17) | '# of 2-level bonding patterns', | DLI(18) | '# of 3-level bonding patterns', |
| DLI(19) | '# of fragments', | DLI(20) | '# of aromatic systems', |
| DLI(21) | '# of cyclic fragments', | DLI(22) | '# of linkers', |
| DLI(23) | '# of cap fragments', | DLI(24) | 'maximum SSSR size', |
| DLI(25) | 'maximum cap fragment size', | DLI(26) | 'total number of 3-8 membered rings', |
| DLI(27) | 'total number of 3to8 saturated rings', | DLI(28) | 'total number of 3to8 unsaturated rings', |
